# Mediating Mechanisms of the Incredible Years Teacher Classroom Management Program

**DOI:** 10.3389/fpsyg.2020.555442

**Published:** 2020-09-29

**Authors:** Håvard Horndalen Tveit, May Britt Drugli, Sturla Fossum, Bjørn Helge Handegård, Christian A. Klöckner, Frode Stenseng

**Affiliations:** ^1^The Regional Centre for Child and Youth Mental Health and Child Welfare – Central Norway, Norwegian University of Science and Technology (NTNU), Trondheim, Norway; ^2^Centre for the Study of Educational Practice (SePu), Innlandet University College, Hamar, Norway; ^3^The Regional Centre for Child and Youth Mental Health and Child Welfare – North, UiT The Arctic University of Norway, Tromsø, Norway; ^4^Department of Psychology, Norwegian University of Science and Technology (NTNU), Trondheim, Norway; ^5^Department of Education and Lifelong Learning, Norwegian University of Science and Technology (NTNU), Trondheim, Norway; ^6^Queen Maud University College, Trondheim, Norway

**Keywords:** Incredible Years, intervention, multilevel mediation, child–teacher, relationship

## Abstract

This study examined whether, and the extent to which, the Incredible Years Teacher Classroom Management program predicted positive development of children’s emotional, behavioral, and social adjustment through changes in the child–teacher relationship. Using data from a longitudinal quasi-experimental intervention trial with a matched control condition, including 1,085 children (49.7% girls, mean_age_ = 4.22 years; SD_age_ = 0.88 years), the potential associations were tested by means of multilevel path modeling. The mediation model demonstrated that (1) children in the intervention condition achieved more favorable changes in the child–teacher relationship than the control condition; (2) changes in the child–teacher relationship were associated with changes in the target outcomes; and (3) the intervention effects were mediated via changes in the child–teacher relationship.

## Introduction

Research has documented that emotional, behavioral, and social development is intrinsically linked to early childhood attachment patterns with caregivers (Sabol and Pianta, 2012; Cantor et al., 2019). As adaptive and maladaptive behaviors are hypothesized to spread within and between domains over time (Bornstein et al., 2010; Masten and Cicchetti, 2010), primary caregivers play a unique role in setting the developmental trajectory of the children in their care. To promote adaptive development, many early-years interventions, including Early Risers (August et al., 2001), Tools of the Mind (Bodrova and Deborah, 2007), and Preschool Paths/Paths programs (Domitrovich et al., 1999), are based on the assertion that childcare and school teachers are catalyst of change in children’s lives. The premise of such interventions is that promoting teacher caregiver competence will nurture more affectionate and intimate child–teacher relationships with less conflictual interactions, thereby creating an environment that stimulates the children’s emotional, behavioral, and social development.

The Incredible Years Teacher Classroom Management (IY TCM) program (Webster-Stratton et al., 2011) is another intervention adhering to this rationale and forms the case study for this article. Despite being extensively examined with various target populations and in multiple countries (Webster-Stratton et al., 2011; Pidano and Allen, 2015) and frequently shown to achieve favorable changes in indicators of children’s mental health and social functioning, empirical support for the proposed causal model of change is scarce. Hence, the present study investigates the potential mediating role of child–teacher relationships in the IY TCM program on three target outcomes in a longitudinal intervention including 1,085 children aged 3–6 years.

The IY TCM program is a universal preventive intervention for childcare and school teachers designed to prevent emotional and behavioral difficulties and promote social competence for children aged 3 through 8 years (Webster-Stratton et al., 2011). The content and principles of the intervention are most notably, but not exclusively, grounded in the works of Ainsworth (1969), Bowlby (1982), Patterson (Patterson et al., 1992), and Bandura (2001), emphasizing secure attachments, nurturing interactions, and learned behavior through observations. Based on the aforementioned theoretical rationale, the intention of the IY TCM intervention is to improve essential caregiver competence skills (e.g., increased sensitivity toward the children’s emotional, social, and physical needs; decreased use of critical and harsh discipline; predictable routines; appropriate limit-setting; respect for the child’s autonomy) as the means to nurture better child–teacher relationships (Webster-Stratton and Reid, 2008). The research literature on the IY TCM program supports its intentions, with favorable changes in the targeted teacher-related outcomes of teacher caregiver competence (Shernoff and Kratochwill, 2007; Raver et al., 2008; Hutchings et al., 2013) and child–teacher relationship (Aasheim et al., 2018; Tveit et al., 2019), as well as the addressed child-related outcomes of emotional and behavioral difficulties (Baker-Henningham et al., 2012; Fossum et al., 2017; Ford et al., 2019) and social competence (Reinke et al., 2018; Aasheim et al., 2019). In addition, several studies have looked at indicators of classroom management, reporting moderate to high effects positive classroom climate (Murray et al., 2018) and teacher sensitivity (Raver et al., 2008), emphasizing the broader implications of the program.

Beyond the preliminary stages of research, once it has been demonstrated whether, to what extent, and for which populations an intervention succeeds in creating favorable changes, questions as to *how* interventions exert their effects become essential—in other words, it is necessary to identify the *facilitating* components of the intervention (Preacher, 2015; O’Rourke and MacKinnon, 2018). A greater understanding of these components could potentially guide further theory development and, at a minimum, indicate how interventions may be improved to achieve greater impact. The latter is of particular interest to early-years interventions, as several meta-analyses have found that interventions, including the IY TCM, targeting emotional, behavioral, and social outcomes often only produce small to moderate effects (Blok et al., 2005; Werner et al., 2016; Egert et al., 2018).

The present study aims at providing some preliminary findings regarding the proposed mediating mechanisms of the IY TCM program, namely, that promoting teachers’ caregiver competence will nurture more affectionate and less conflictual child–teacher relationships, which, in effect, will stimulate the children’s emotional, behavioral, and social adjustment. Such a mediating association is embedded in the theoretical models, and interventions based on this premise have indeed achieved favorable changes in the target *outcome*. Nevertheless, the models’ *mediating* processes have been left unexamined. Therefore, using data from a longitudinal quasi-experimental intervention study with a matched control condition, including 1,085 children aged 3–6 years, we tested the proposed mediating mechanisms using multilevel path modeling. More specifically, this article investigated whether, and the extent to which, the intervention effects on emotional, behavioral, and social adjustment were mediated by changes in child–teacher closeness and child–teacher conflict.

## Materials and Methods

### Trial Procedures

Before the study commenced, the study protocol and procedures were approved by the Regional Committee for Medical and Health Research Ethics. Information about the IY TCM program and research study was presented to the childcare staff and parents. Parents were informed of the possibility to withdraw consent at any time without reprisal, and childcare teachers were not allowed to complete the assessments before written parental consent was given. The intervention program was implemented free of charge, and the childcare centers were financially compensated for the time the childcare teachers had to spend completing the assessments. Neither the teachers, nor the children received any payment for their participation, as to avoid biasing the participants. The intervention procedures were conducted by IY Norway to ensure high levels of fidelity, whereas the research trial was conducted by researchers with no affiliation to the IY organization. All data were anonymized in accordance with national regulations.

The trial used continuous enrollment of childcare centers of a 5-year duration, lasting from autumn 2009 to autumn 2013. The baseline assessment was conducted in October each year, 1–3 weeks before initiation, and the follow-up assessment took place 1–3 weeks after the intervention period was finished in the end of June. Approximately 9 months passed between the assessments, during which the IY TCM program was implemented in the intervention condition. Over the same period, the control condition carried on as usual.

### Sample Recruitment

In Norway, 97.1% of all children aged 3–6 years attend childcare centers (Statistics Norway, 2019, March), and 96.4% of the children are registered for 41 h or more per week at their unit. Children from linguistic and cultural minorities have an attendance rate of 82.2%, and national regulations ensure affordable childcare for all, with a funding scheme based on the parent’s income. About 50% of all childcare centers in Norway are under private ownership, but as with publicly owned childcare units, they must act in accordance to the Kindergarten Act (Act no. 64 2005). The key features of the Act are one caregiver per six children and one pedagogical leader per 14–18 children, and all pedagogical leaders must be educated childcare teacher. Furthermore, the Framework Plan (a section included in the Kindergarten Act) provide guidelines in relation to values, learning objectives, and pedagogical undertaking that each childcare center must accommodate.

Childcare centers were invited by IY Norway to participate in the trial. To be eligible to participate in the intervention condition, all units at the childcare center had to participate, at least 80% of the staff had to approve of participation, and the staff were prohibited from attending any other training modules during the study period. Childcare centers in the control condition were strategically matched with childcare centers in the intervention condition with respect to geographical location and number of children enrolled. Childcare centers in the matched control condition received the intervention the following year free of charge.

**FIGURE 1:**
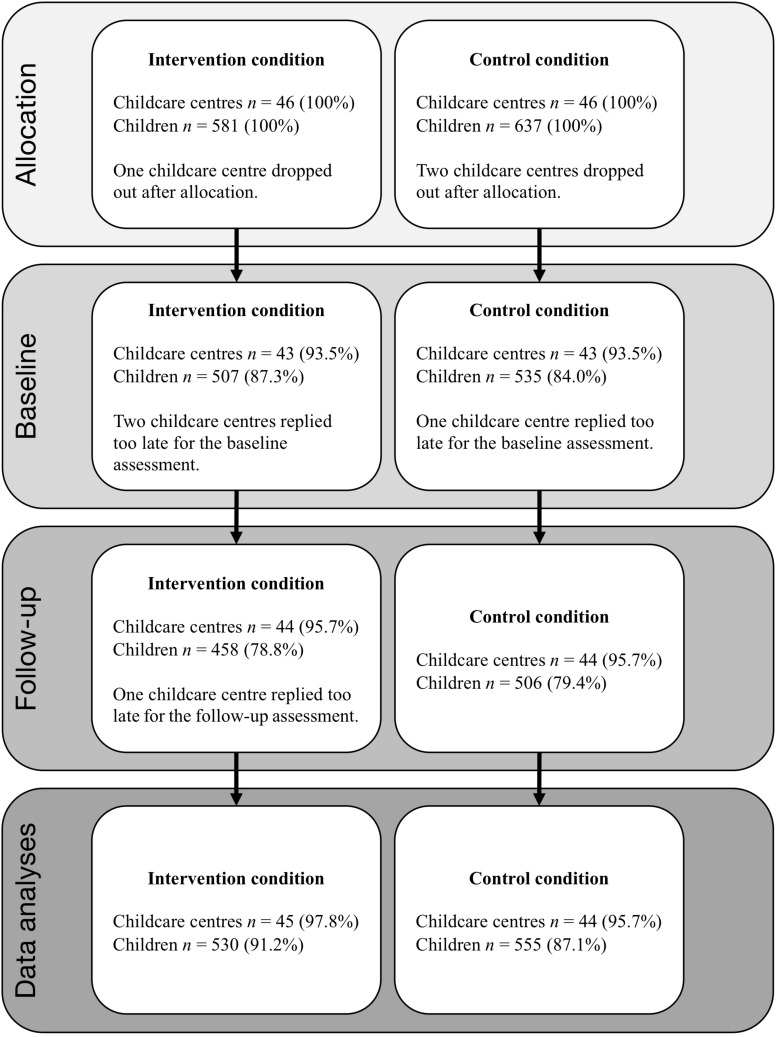
Flowchart of childcare centers and participants.

Sample size estimation was conducted *a priori* based on a two-sided group comparison on the outcome measures. A stepwise approach was utilized, accounting for the shared variance inherent in the clustering of children within childcare centers, by first estimating a naive sample size and then adjusting for the design effect to achieve a robust sample size. The design effect, which takes the intraclass correlation coefficient (ICC) and the average cluster size into consideration (Maas and Hox, 2005), provides an estimate of how much larger the robust sample size needs to be relative to the naive sample size. With the true ICC unknown, an ICC of.20 was chosen as recommended for behavioral measures (Scherbaum and Ferreter, 2009). To achieve a balance between the cost of sampling at each level, the unique contribution of each level-1 participant, and the workload on each teacher, the cluster size was set to seven. The robust sample size indicated that data collection could conclude once 46 childcare centers were included in each condition. Through a randomized selection process, seven children from each unit were chosen to participate in the trial, resulting in 581 children in the intervention condition and 637 children in the control condition, because of variation in the numbers of units at each childcare center (Figure 1).

### Participants

#### Children

At baseline, the total sample of 1,218 children (50.3% boys) averaged an age of 4.22 years (*SD* = 0.88 years), with 581 children [48.9% boys, 4.16 years (*SD* = 0.89)] in the intervention condition and 637 children [51.7% boys, 4.27 years (*SD* = 0.85)] in the control condition. A total of 90.4% of the children spent 30 h of more per week at the childcare center, close to the national average of 91.8% for the respective age group (Statistics Norway, 2019). Forty-seven children had special educational needs, similar to the 4.2% national average (Statistics Norway, 2019, March).

#### Childcare Centers

The 92 childcare centers included in the study represented 10 of 19 counties in Norway, with an equal combination of municipal and private ownership. Thirty-three percent of the childcare centers had more than 49 children enrolled, and the average unit group size in both conditions was 19 children. Among the childcare staff, 9.2% were male, similar to the national average (Statistics Norway, 2019, March), with most teachers reporting knowing the children either well or reasonably well at baseline.

### Intervention Procedures

The intervention procedures comply with the IY TCM manual and are described in detail elsewhere (Webster-Stratton et al., 2011). In brief, the intervention is structured around six workshop sessions, each lasting approximately 7 h, held once every fourth week during the 9-month intervention period, with the entire childcare staff at the respective units participating. The workshop sessions are organized by two experienced, IY-certified group leaders. The first session details why caregiver competence is key to creating warm, nurturing child–teacher relationships, while successive sessions expand on how child–teacher relationships can help stimulate the children’s emotional, behavioral, and social competence. The sessions emphasize hands-on experience, including instructional video vignettes, discussions, sharing of experiences, role-play, and self-reflection. Furthermore, the childcare staff receive reading assignments and are instructed to practice the principles when back at their units. In addition, group leaders provide one-on-one guidance throughout the intervention period, and teachers report on their experiences at the following workshop session to stimulate discussion and learn from one another.

### Measures

#### Child–Teacher Relationship

The child–teacher closeness and child–teacher conflict scales of the Student-Teacher Relationship Scale, Short Form (STRS) (Pianta, 2001) were used to assess the characteristics of the two dimensions of the child–teacher relationship. The teachers evaluated the extent to which each of 15 statements reflected their relationship to a particular child on a 5-point Likert scale. The psychometric properties are considered satisfactory (Pianta, 2001), with the factorial validity of the two-dimensional Short Form outperforming the original three-dimensional solution (the third dimension, *dependency*, is excluded in the short form) in Norwegian samples (Drugli and Hjemdal, 2013). The trial data showed Cronbach α values of 0.80 and 0.81 for the conflict scale (seven items) and 0.80 and 0.78 for the closeness scale (eight items) at baseline and follow-up, respectively.

#### Emotional and Behavioral Difficulties

The internalizing and externalizing scales from the Caregiver–Teacher Report Form 1.5-5 (C-TRF) (Achenbach and Rescorla, 2000) were used to evaluate the children’s emotional and behavioral difficulties, respectively. The teachers rated the occurrences of problematic situations over the last 6 months from 0 = “not true” to 2 = “very true.” The internalizing scale consists of four subdimensions (emotionally reactive, anxious/depressed, somatic complaints, and withdrawn), while the externalizing scale consists of two dimensions (attention problems and aggressive behavior), with the summed scores of the subdimensions providing the total score of the two scales, respectively. The psychometric properties of the internalizing and externalizing scales of the C-TRF are very good, whereas the subdimensions (particularly somatic complaints) are less satisfactory (Achenbach et al., 2008). The trial data showed Cronbach α at 0.83 and 0.82 for the internalizing scale and 0.94 and 0.95 for the externalizing scale at baseline and follow-up, respectively. Because of the multidimensionality of the tests, α is in addition reported at the subdimensional level: emotionally reactive (0.66 and 0.63), anxious/depressed (0.60 and 0.62), somatic complaints (0.45 and 0.34), withdrawn (0.80 and 0.74), attention problems (0.87 and 0.86), and aggressive behavior (0.93 and 0.93). Despite the low α scores of the somatic complaints dimension, the dimension is still included in the total internalizing score due to fidelity.

#### Social Competence

The social competence scale of the Social Competence and Behaviour Evaluation for Teachers (SCBE) (LaFreniere and Dumas, 1995) was employed to measure the strengths and positive characteristics of the children. The SCBE is a 40-item, 6-point Likert rating scale, with which the teachers evaluated the children’s behavior in typical childcare setting situations. Higher scores on the SCBE indicate that the child has higher levels of social maturity and competence, exhibits prosocial behavior, and is self-confident. The SCBE shows satisfactory fit in regard to convergent, discriminative, and predictive validity (Humphrey et al., 2011). Internal consistency was 0.97 at both baseline and follow-up.

### Statistical Analyses

#### Preliminary Analyses

As a multisite trial, with a sample of children nested within teachers, the shared variance is expected to be relatively high within each cluster, which—if not accounted for—can bias the standard error and increase the probability of type I error (Musca et al., 2011). The ICC was estimated from the baseline measures with linear mixed-model analysis using the unconditional means model (Singer and Willett, 2003) to determine whether the nesting was to be included in the mediation model. Next, all available demographical variables were regressed on the mediators and outcome measures to examine if any of the variables should be included as covariates in the mediation model, the criteria being a two-sided *p*-value of less than 0.05. Finally, mixed models were run to examine any baseline differences between the intervention and control condition, as well as between participants with complete data and participants lost to follow-up.

#### Missing Responses

In the case of missing items on either the outcome or proposed mediating variables, the procedures described in the respective manuals were followed. According to all three manuals, if the number of missing items is below a given threshold, the recommended procedure is mean imputation within participant and scale; otherwise, the scale is deemed invalid and left out of the analyses. The threshold is one item per scale of the STRS, eight of the total 99 items of the C-TRF, and four items of the SCBE.

#### Model Specifications

**FIGURE 2:**
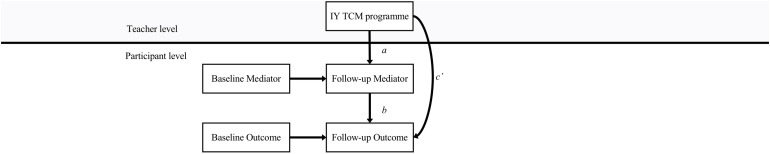
General outline of the mediation model.

A multilevel path model was fitted to examine the proposed mediation of the IY TCM program in *R* (R Core Team, 2019) with the *Lavaan* package (Rosseel, 2012). The structural equation framework was applied, to allow for the flexibility when repeated measures and multiple mediators are included, in addition to providing fit indices for model comparisons (Preacher et al., 2010). Figure 2 indicates how each mediator and outcome are modeled, with autoregressive paths between baseline and follow-up; paths between the intervention, mediator, and outcome at follow-up; and the multilevel nesting. However, both mediators and all three outcomes were fitted in the same model due to high correlation between mediators on the one hand, and outcomes on the other; thus, the full model included two *a* paths, six *b* paths, and three *c*’ paths.

The model is seen structured as a 2-1-1 design (Krull and MacKinnon, 2001), indicating that variation in the predictor could only exist between teachers, whereas mediators and outcome variables could vary between participants. In line with the theoretical rationale of the IY TCM program, the teacher-level intervention would be expected to impact the participant-level outcomes via participant-level mediators; hence, we tested the cross-level mediation paths explicitly (Pituch and Stapleton, 2012). Statistical considerations provided further justification for excluding the cluster-level mediation paths, as the limited level-2 sample size could reduce both power and precision in cases where no contextual effects are present (Pituch and Stapleton, 2012).

The models were estimated with full information maximum likelihood (FIML) to avoid any loss of information, as FIML has been shown to outperform multiple imputation in longitudinal multilevel data (Larsen, 2011). All relevant fit measures are reported.

#### Test of Mediation

In the mediation model outlined, the point estimates for the mediated effects are calculated according to the *a* ^∗^ *b* product approach (Zigler and Ye, 2019): *a* represents the mean treatment effect on the mediators between the conditions, whereas *b* expresses the mediators’ impact on the outcomes when controlling for the treatment, and *c*’, the direct effect of the treatment on the outcomes when controlled for the by the mediators. Confidence intervals for the estimated effects were constructed using 10,000 Monte Carlo simulations (Selig and Preacher, 2008; Preacher and Selig, 2012).

## Results

The unconditional means model confirmed that the hierarchical structure of the data needed to be accounted for (Table 1); thus, the nesting was included in all further analyses. A series of linear mixed models revealed that no demographical measures predicted any of the mediators or outcomes, neither did any of the baseline measures differ between the conditions; hence, no demographical covariates were included in the mediation analysis.

**TABLE 1:** Means, standard error, and ICC for mediator and outcome by condition and time.

	Intervention	Control	ICC
**Baseline**			
Child–teacher closeness	29.39 ± 0.26 (*n* = 507, 87.3%)	30.06 ± 0.25 (*n* = 535, 84.0%)	0.278
Child–teacher conflict	12.34 ± 0.30 (*n* = 507, 87, 3%)	12.21 ± 0.30 (*n* = 535, 84.0%)	0.217
Emotional difficulties	2.97 ± 0.27 (*n* = 393, 67.6%)	2.92 ± 0.24 (*n* = 477, 74.9%)	0.188
Behavioral difficulties	5.53 ± 0.50 (*n* = 393, 67.6%)	4.82 ± 0.45 (*n* = 477, 74.9%)	0.122
Social competence	94.4 ± 1.98 (*n* = 502, 86.4%)	94.2 ± 1.94 (*n* = 523, 82.1%)	0.211
**Follow-up**			
Child–teacher closeness	31.28 ± 0.26 (*n* = 458, 78.8%)	30.73 ± 0.25 (*n* = 506, 79.4%)	0.254
Child–teacher conflict	11.39 ± 0.30 (*n* = 458, 78.8%)	12.23 ± 0.30 (*n* = 506, 79.4%)	0.218
Emotional difficulties	2.51 ± 0.27 (*n* = 375, 64.5%)	2.90 ± 0.24 (*n* = 462, 72.5%)	0.160
Behavioral difficulties	4.35 ± 0.50 (*n* = 375, 64.5%)	4.86 ± 0.45 (*n* = 462, 72.5%)	0.134
Social competence	89.47 ± 2.00 (*n* = 451, 77.6%)	93.71 ± 1.95 (*n* = 496, 77.9%)	0.234

*The number in the parentheses refers to the number of participants with valid scores and the percent of the total sample within the condition.*

As shown in Figure 1, three childcare centers dropped out after allocation (one in the intervention and two in the control condition), which gave a total of 45 childcare centers, including 530 children, in the intervention condition, and 44 childcare centers, with 555 children, in the control. Furthermore, two childcare centers in the intervention condition and one childcare center in the control replied too late to be included in the baseline assessment, and finally, one childcare center replied too late for the follow-up assessment in the intervention condition. Because of technical issues with the electronic questionnaire, a number of items measuring emotional and behavioral difficulties were not included in the first round of enrollment of childcare centers, leading to a higher percentage of participants scoring above the accepted threshold for the CTRF. The total number of participants with valid scores is reported in Table 1.

Group-by-attrition analyses found that participants in the control condition lost to follow-up had lower scores than did participants with complete data within the same condition on child–teacher closeness (−1.92; 95% CI, −3.21 to −0.65), higher scores on child–teacher conflict (1.67; 95% CI, 0.11–3.22), and higher scores on emotional difficulties (1.81; 95% CI, 0.48–3.12) at baseline. No other differences were significant.

The mediation analysis was fitted as one multilevel path model. Model fit parameters confirmed a good model fit [comparative fit index = 0.989, Tucker–Lewis Index = 0.901, root mean square error of approximation = 0.077, standardized root mean square residual (SRMR) within = 0.024, SRMR between = 0.28].

First, as seen in Table 2, the children in the intervention condition had a larger improvement in child–teacher closeness (*a*_1_: β = 0.953; 95% CI, 0.331–1.574) than children in the control condition, and a greater reduction in child–teacher conflict (*a*_2_: β = −1.377; 95% CI, −2.128 to −0.626) after the 9-month intervention period.

**TABLE 2:** Multilevel path model.

	Point estimates			95% CI	
	Unstandardized	Standardized	SE	Lower	Upper
**Child–teacher closeness as mediator**					
(*a*_1_) Int. → closeness	0.953	0.255	0.317	0.331	1.574
(*b*_1_) Closeness → emo. diff.	–0.166	–0.152	0.033	–0.230	–0.102
(*b*_2_) Closeness → behav. diff.	–0.202	–0.090	0.053	–0.306	–0.099
(*b*_3_) Closeness → soc. comp.	2.380	0.300	0.207	1.974	2.786
(*a*_1_ * *b*_1_) Mediated on emo. diff.	–0.158	–0.039	0.061	–0.291	–0.051
(*a*_1_ * *b*_2_) Mediated on behav. diff.	–0.193	–0.023	0.082	–0.377	–0.055
(*a*_1_ * *b*_3_) Mediated on soc. comp.	2.268	0.076	0.784	0.745	3.865
**Child–teacher conflict as mediator**					
(*a*_2_) Int. → conflict	–1.377	–0.298	0.383	–2.128	–0.626
(*b*_4_) Conflict → emo. diff.	0.247	0.305	0.030	0.189	0.305
(*b*_5_) Conflict → behav. diff.	0.607	0.362	0.047	0.515	0.699
(*b*_6_) Conflict → soc. comp.	–1.511	–0.256	0.177	–1.858	–1.165
(*a*_2_ * *b*_4_) Mediated on emo. diff.	–0.340	–0.091	0.104	–0.557	–0.145
(*a*_2_ * *b*_5_) Mediated on behav. diff.	–0.836	–0.108	0.246	–1.338	–0.367
(*a*_2_ * *b*_6_) Mediated on soc. comp.	2.081	0.076	0.624	0.903	3.372
**Direct effects when controlled for by mediation paths**					
(*c*’_1_) Int. → emo. diff.	–0.063	–0.021	0.229	–0.512	0.386
(*c*’_2_) Int. → behav. diff.	–0.285	–0.055	0.443	–1.153	0.583
(*c*’_3_) Int. → soc. comp.	0.530	0.014	2.477	–4.324	5.384

*Int., intervention; closeness, child–teacher closeness; emo. diff, emotional difficulties; behav. diff, behavioral difficulties; soc. comp., social competence; conflict, child–teacher conflict.*

Second, changes in child–teacher closeness were associated with emotional difficulties (*b*_1_: β = −0.166; 95% CI, −0.230 to −0.102), behavioral difficulties (*b*_2_: β = −0.202; 95% CI, −0.306 to −0.099), and social competence (*b*_3_: β = 2.380; 95% CI, 1.974–2.786). Likewise, changes in child–teacher conflict were associated with emotional difficulties (*b*_4_: β = 0.247; 95% CI, 0.189–0.305), behavioral difficulties (*b*_5_: β = 0.607; 95% CI, 0.515–0.699), and social competence (*b*_6_: β = −1.511; 95% CI, −1.858 to −1.165).

Finally, the mediated effect of child–teacher closeness on emotional difficulties (*a*_1_^∗^ *b*_1_) was −0.158 (95% CI, −0.291 to −0.051), closeness on behavioral difficulties (*a*_1_^∗^ *b*_2_) was −0.193 (95% CI, −0.377 to −0.055), and closeness on social competence (*a*_1_^∗^ *b*_3_) was 2.268 (95% CI, 0.745–3.865). The mediated effect of child–teacher conflict on emotional difficulties (*a*_2_^∗^ *b*_4_) was −0.340 (95% CI, −0.557 to −0.145), conflict on behavioral difficulties (*a*_2_^∗^ *b*_5_) was −0.836 (95% CI, −1.338 to −0.367), and conflict on social competence (*a*_2_^∗^ *b*_6_) was 2.081 (95% CI, 0.903–3.372). The direct effects of the intervention on the outcome measures when controlled for by the mediation paths (*c*’_1_, *c*’_2_, and *c*’_3_) were all non-significant.

## Discussion

In this 9-month quasi-experimental intervention study, using a large sample of children from childcare centers in Norway, we investigated the proposed mediating mechanisms underlying the IY TCM program, namely, child–teacher relationships. The IY TCM program states that by improving the teachers’ caregiver competence, children will form more affectionate and less conflictual child–teacher relationships, stimulating, in effect, the children’s emotional, behavioral, and social development. The data indeed supported the conjecture. First, as demonstrated in previous studies, the intervention condition achieved more favorable changes in child–teacher closeness and child–teacher conflict than the control condition (*a* paths). Second, changes in child–teacher closeness and child–teacher conflict were associated with changes in emotional, behavioral, and social adjustment (*b* paths). Finally, the intervention effects were significantly mediated through changes in child–teacher relationship (*a* ^∗^ *b* paths). Furthermore, the direct effects were non-significant when controlled for by the mediators (*c*’ paths), indicating complete mediation. The strengths of associations for the mediated effects were most distinct through child–teacher closeness and conflict to social competence, whereas the mediated effects to emotional and behavioral difficulties were, although significant, less apparent.

The findings in this article corroborate the theoretical rationale underlying the IY TCM program, indicating that child–teacher relationships are the key mechanism transmitting the effects of the intervention on the children’s emotional, behavioral, and social adjustment. Practitioners of the IY TCM program might thus expect to achieve greater effects by further emphasizing the importance of promoting child–teacher closeness and reducing conflict as a means to healthy child development. The changes manifesting, not only in the children’s behavior, but also in the childcare teachers approach toward the children, are a further aspect to appreciate. Over the duration of 9 months, the teachers have seemingly improved their caregiving skills, thus creating an environment that is better suited for positive and thriving experiences for both child and teacher. Should the changes in caregiving skills persist over time, as some studies indicate (Raver et al., 2008; Carlson et al., 2011), the beneficiaries of the IY TCM program are not limited to only the children currently attending the childcare center, but also for children attending the childcare center in the future as well.

It could be argued that the core focus of the intervention is on creating *positive* change, by promoting positive caregiver skills and sensitivity toward the child, rather than attempting to *decrease* negative caregiving. Promoting child–teacher interaction skills, for instance, comprises a substantial part of the workshop material and seems essential for change to manifest in the first place. Yet, in studies that include measures of both positive and negative caregiver skills, the reduction of negative caregiving is shown to have a stronger influence on the children’s behavior than increasing positive caregiving (Hanisch et al., 2014). Granted, resolving conflict, and breaking free form coercive interactions, does receive attention in the IY TCM program, it does so to a lesser degree than the promotion of positive caregiving skills. Perhaps including measures of positive and negative caregiving in future trials could further improve our understanding of the underlying mechanisms of change in the IY TCM program.

A common misconception in mediation analysis relates to causal interpretation of results, and readers ought to be aware that the results do not provide evidence of causality as temporal precedence was breached (Kazdin, 2007); for claims of causality cause must precede effect (Green et al., 2010), while in this study the mediators and outcomes were measured at the same time. A possible solution to maintain temporal order with only two waves is the “half-longitudinal design” as proposed by Cole and Maxwell (2003). Still, the half-longitudinal design has two important drawbacks; it hinges on the assumption of stationarity of the *b* paths, in addition to not permitting the estimation of direct effects. As participants in this study were young children in a period characterized by rapid development and change, the assumption of stationarity was questionable, while not allowing estimation of direct effects prohibits tests for full or partial mediation, thus limiting the significance of the findings. Furthermore, as a pre-post trial, the potential reciprocal association between the mediators and outcomes could not be modeled as in a trial with additional measurement occasions, conceivably introducing more uncertainty in the estimated mediation paths. Next, the intervention effects are assumed equal for all participants in the mediation model outlined, although heterogeneity within participants is to be expected (Bolger et al., 2019). An important issues is that the multilevel path model tested used manifest variables, rather than latent, which hinges on the assumption that variables are assessed without measurement error, but as Cole and Preacher (2014) point out, this is a unreasonably strong assumption and can create biased point estimates in complex models. Additionally, the study relied on teacher responses exclusively and would have benefitted from other data sources to corroborate, or potentially challenge, the results from this study. Thus, readers should recognize that the results might be biased in terms of response bias (the teacher’s belief in the intervention affected their response) or selective perception bias (the teacher’s understanding of the phenomenon improved rather than the actual phenomenon itself). Finally, Fairchild and McDaniel (2017) have argued that pre-post trials probing mediation provide preliminary insights into the mediating process, but need to be conducted with more rigorous designs to support claims of causality—including manipulation of mediators, additional assessments, and potential confounders. The authors support these suggestions and encourage further investigation.

## Conclusion

To the best of our knowledge, this is the first study to examine the theoretical rationale of the IY TCM program through mediation analysis. The data demonstrated support in favor of the model, with satisfactory fit and significant mediation estimates, as changes in the child–teacher relationship mediated the intervention effects of the children’s emotional, behavioral, and social adjustment. A deeper understanding of the underlying mechanisms of the IY TCM intervention is an important first step toward achieving greater impact, and this study suggests that with further emphasis placed on improving child–teacher closeness and reducing child–teacher conflict, greater intervention effects might manifest, benefitting both childcare teachers and children.

## Data Availability Statement

The datasets presented in this article are not readily available because of a technical delay. However, UiT, The Arctic University of Norway is in the process of making all meta data in research projects available for sharing through the Eutro programme. Requests to access the datasets should be directed to https://uit.no/forskning/forskningsgrupper/gruppe?p_document_id=525017, eutro@helsefak.uit.no.

## Ethics Statement

The studies involving human participants were reviewed and approved by the Regional Committees for Medical and Health Research Ethics. Written informed consent to participate in this study was provided by the participants’ legal guardian/next of kin.

## Author Contributions

HHT conceived the presented idea. HHT, FS, and MBD developed the theoretical framework. HHT, BHH, and CAK performed the statistical analyses. HHT wrote the manuscript with support from FS, MBD, and SF. All authors contributed to the final manuscript and approved it for publication.

## Conflict of Interest

The authors declare that the research was conducted in the absence of any commercial or financial relationships that could be construed as a potential conflict of interest.

## References

[B1] Aasheim M. Drugli M. -B. Reedtz C. Handegård B. H. Martinussen M. (2018). Change in teacher–student relationships and parent involvement after implementation of the incredible years teacher classroom management programme in a regular norwegian school setting. *Br. Educ. Res. J.* 44 1064–1083. 10.1002/berj.3479

[B2] Aasheim M. Reedtz C. Handegård B. H. Martinussen M. Mørch W.-T. (2019). Evaluation of the incredible years teacher classroom management program in a regular norwegian school setting. *Scand. J. Educ. Res.* 63 899–912. 10.1080/00313831.2018.1466357

[B3] Achenbach T. M. Becker A. Döpfner M. Heiervang E. Roessner V. Steinhausen H. C. (2008). Multicultural assessment of child and adolescent psychopathology with ASEBA and SDQ instruments: research findings, applications, and future directions. *J. Child Psychol. Psychiatry* 49 251–275. 10.1111/j.1469-7610.2007.01867.x 18333930

[B4] Achenbach T. M. Rescorla L. A. (2000). *Manual for the ASEBA Preschool Forms & Profiles.* Burlington, VT: University of Vermont.

[B5] Act no. 64 (2005). *The Kindergarten Act.* Oslo: Ministry of Education and Research.

[B6] Ainsworth M. D. (1969). Object relations, dependency, and attachment: a theoretical review of the infant-mother relationship. *Child Dev.* 40 969–1025. 10.2307/1127008 5360395

[B7] August G. J. Realmuto G. M. Hektner J. M. Bloomquist M. L. (2001). An integrated components preventive intervention for aggressive elementary school children: the early risers program. *J. Consult. Clin. Psychol.* 69 614–626. 10.1037/0022-006x.69.4.614 11550728

[B8] Baker-Henningham H. Scott S. Jones K. Walker S. (2012). Reducing child conduct problems and promoting social skills in a middle-income country: cluster randomised controlled trial. *Br. J. Psychiatry* 201 101–108. 10.1192/bjp.bp.111.096834 22500015 PMC3409425

[B9] Bandura A. (2001). Social cognitive theory: an agentic perspective. *Annu. Rev. Psychol.* 52 1–26. 10.1146/annurev.psych.52.1.1 11148297

[B10] Blok H. Fukkink R. Gebhardt E. Leseman P. (2005). The relevance of delivery mode and other programme characteristics for the effectiveness of early childhood intervention. *Int. J. Behav. Dev.* 29 35–47. 10.1080/01650250444000315

[B11] Bodrova E. Deborah J. L. (2007). *Tools of the Mind: The Vygotskian Approach to Early Childhood Education*, 2nd Edn Columbus, OH: Pearson.

[B12] Bolger N. Zee K. S. Rossignac-Milon M. Hassin R. R. (2019). Causal processes in psychology are heterogeneous. *J. Exp. Psychol. Gen.* 148 601. 10.1037/xge0000558 30973259

[B13] Bornstein M. H. Hahn C.-S. Haynes O. M. (2010). Social competence, externalizing, and internalizing behavioral adjustment from early childhood through early adolescence: developmental cascades. *Dev. Psychopathol.* 22 717–735. 10.1017/s0954579410000416 20883577 PMC3412561

[B14] Bowlby J. (1982). *Attachment and Loss: Attachment.* New York, NY: Basic Books.

[B15] Cantor P. Osher D. Berg J. Steyer L. Rose T. (2019). Malleability, plasticity, and individuality: how children learn and develop in context. *Appl. Dev. Sci.* 23 307–337. 10.1080/10888691.2017.1398649

[B16] Carlson J. S. Tiret H. B. Bender S. L. Benson L. (2011). The influence of group training in the incredible years teacher classroom management program on preschool teachers’ classroom management strategies. *J. Appl. Sch. Psychol.* 27 134–154. 10.1080/15377903.2011.565277

[B17] Cole D. A. Maxwell S. E. (2003). Testing mediational models with longitudinal data: questions and tips in the use of structural equation modeling. *J. Abnormal Psychol.* 112 558. 10.1037/0021-843x.112.4.558 14674869

[B18] Cole D. A. Preacher K. J. (2014). Manifest variable path analysis: potentially serious and misleading consequences due to uncorrected measurement error. *Psychol. Methods* 19 300. 10.1037/a0033805 24079927

[B19] Domitrovich C. E. Greenberg M. T. Kusché C. Cortes R. (1999). *Manual for the Preschool PATHS Curriculum.* South Deerfield, MA: Channing Bete Company.

[B20] Drugli M. B. Hjemdal O. (2013). Factor structure of the student–teacher relationship scale for norwegian school-age children explored with confirmatory factor analysis. *Scand. J. Educ. Res.* 57 457–466. 10.1080/00313831.2012.656697

[B21] Egert F. Fukkink R. G. Eckhardt A. G. (2018). Impact of in-service professional development programs for early childhood teachers on quality ratings and child outcomes: a meta-analysis. *Rev. Educ. Res.* 88 401–433. 10.3102/0034654317751918

[B22] Fairchild A. J. McDaniel H. L. (2017). Best (but oft-forgotten) practices: mediation analysis. *Am. J. Clin. Nutr.* 105 1259–1271. 10.3945/ajcn.117.152546 28446497 PMC5445681

[B23] Ford T. Hayes R. Byford S. Edwards V. Fletcher M. Logan S. (2019). The effectiveness and cost-effectiveness of the incredible years§teacher classroom management programme in primary school children: results of the stars cluster randomised controlled trial. *Psychol. Med.* 49 828–842. 10.1017/s0033291718001484 30017006 PMC6425365

[B24] Fossum S. Handegård B. H. Drugli M. B. (2017). The incredible years teacher classroom management programme in kindergartens: effects of a universal preventive effort. *J. Child Fam. Stud.* 26 2215–2223. 10.1007/s10826-017-0727-3

[B25] Green D. P. Ha S. E. Bullock J. G. (2010). Enough already about “black box” experiments: studying mediation is more difficult than most scholars suppose. *Ann. Am. Acad. Polit. Soc. Sci.* 628 200–208. 10.1177/0002716209351526

[B26] Hanisch C. Hautmann C. Plück J. Eichelberger I. Döpfner M. (2014). The prevention program for externalizing problem behavior (PEP) improves child behavior by reducing negative parenting: analysis of mediating processes in a randomized controlled trial. *J. Child Psychol. Psychiatry* 55 473–484. 10.1111/jcpp.12177

[B27] Humphrey N. Kalambouka A. Wigelsworth M. Lendrum A. Deighton J. Wolpert M. (2011). Measures of social and emotional skills for children and young people: a systematic review. *Educ. Psychol. Meas.* 71 617–637. 10.1177/0013164410382896

[B28] Hutchings J. Martin-Forbes P. Daley D. Williams M. E. (2013). A randomized controlled trial of the impact of a teacher classroom management program on the classroom behavior of children with and without behavior problems. *J. School Psychol.* 51 571–585. 10.1016/j.jsp.2013.08.001 24060060

[B29] Kazdin A. E. (2007). Mediators and mechanisms of change in psychotherapy research. *Annu. Rev. Clin. Psychol.* 3 1–27. 10.1146/annurev.clinpsy.3.022806.091432 17716046

[B30] Krull J. L. MacKinnon D. P. (2001). Multilevel modeling of individual and group level mediated effects. *Multi. Behav. Res.* 36 249–277. 10.1207/s15327906mbr3602_06 26822111

[B31] LaFreniere P. J. Dumas J. E. (1995). *Social Competence and Behavior Evaluation, Preschool Edition (SCBE).* Los Angeles, CA: Western Psychological Services.

[B32] Larsen R. (2011). Missing data imputation versus full information maximum likelihood with second-level dependencies. *Struct. Equat. Model. A Multidiscip. J.* 18 649–662. 10.1080/10705511.2011.607721

[B33] Maas C. J. M. Hox J. J. (2005). Sufficient sample sizes for multilevel modeling. *Methodology* 1 86–92. 10.1027/1614-2241.1.3.85

[B34] Masten A. S. Cicchetti D. (2010). Developmental cascades. *Dev. Psychopathol.* 22 491–495. 10.1017/s0954579410000222 20576173

[B35] Murray D. W. Rabiner D. L. Kuhn L. Pan Y. Sabet R. F. (2018). Investigating teacher and student effects of the incredible years classroom management program in early elementary school. *J. School Psychol.* 67 119–133. 10.1016/j.jsp.2017.10.004 29571528

[B36] Musca S. C. Kamiejski R. Nugier A. Méot A. Er-Rafiy A. Brauer M. (2011). Data with hierarchical structure: impact of intraclass correlation and sample size on type-I error. *Front. Psychol.* 2:74. 10.3389/fpsyg.2011.00074 21687445 PMC3110485

[B37] O’Rourke H. P. MacKinnon D. P. (2018). Reasons for testing mediation in the absence of an intervention effect: a research imperative in prevention and intervention research. *J. Stud. Alcohol Drugs* 79 171–181. 10.15288/jsad.2018.79.171 29553343 PMC6019768

[B38] Patterson G. R. Reid J. B. Dishion T. J. (1992). *Antisocial Boys.* Eugene, OR: Castalia Press.

[B39] Pianta R. C. (2001). *Student-Teacher Relationship Scale: Professional Manual.* Odessa, FL: Psychological Assessment Resources.

[B40] Pidano A. E. Allen A. R. (2015). The incredible years series: a review of the independent research base. *J. Child Fam. Stud.* 24 1898–1916. 10.1007/s10826-014-9991-7

[B41] Pituch K. A. Stapleton L. M. (2012). Distinguishing between cross- and cluster-level mediation processes in the cluster randomized trial. *Sociol. Methods Res.* 41 630–670. 10.1177/0049124112460380

[B42] Preacher K. J. (2015). Advances in mediation analysis: a survey and synthesis of new developments. *Annu. Rev. Psychol.* 66 825–852. 10.1146/annurev-psych-010814-015258 25148853

[B43] Preacher K. J. Selig J. P. (2012). Advantages of Monte Carlo confidence intervals for indirect effects. *Commun. Methods Meas.* 16 77–98.

[B44] Preacher K. J. Zyphur M. J. Zhang Z. (2010). A general multilevel SEM framework for assessing multilevel mediation. *Psychol. Methods* 15 209–233. 10.1037/a0020141 20822249

[B45] R Core Team. (2019)). *R: A Language and Environment for Statistical Computing.* Vienna: R Foundation for Statistical Computing. 10.1037/a0020141

[B46] Raver C. C. Jones S. M. Li-Grining C. P. Metzger M. Champion K. M. Sardin L. (2008). Improving preschool classroom processes: preliminary findings from a randomized trial implemented in head start settings. *Early Childhood Res. Quart.* 23 10–26. 10.1016/j.ecresq.2007.09.001 18364994 PMC2274905

[B47] Reinke W. M. Herman K. C. Dong N. (2018). The incredible years teacher classroom management program: outcomes from a group randomized trial. *Prev. Sci.* 19 1043–1054. 10.1007/s11121-018-0932-3 30022357

[B48] Rosseel Y. (2012). Lavaan: an R package for structural equation modeling. *J. Statist. Softw.* 48 1–36. 10.18637/jss.v048.i02

[B49] Sabol T. J. Pianta R. C. (2012). Recent trends in research on teacher–child relationships. *Attach. Hum. Dev.* 14 213–231. 10.1080/14616734.2012.672262 22537521

[B50] Scherbaum C. A. Ferreter J. M. (2009). Estimating statistical power and required sample sizes for organizational research using multilevel modeling. *Org. Res. Methods* 12 347–367. 10.1177/1094428107308906

[B51] Selig J. P. Preacher K. J. (2008). Monte Carlo Method For Assessing Mediation: An Interactive Tool For Creating Confidence Intervals For Indirect Effects. Available online at: http://quantpsy.org/ (accessed February 20, 2020).

[B52] Shernoff E. S. Kratochwill T. R. (2007). Transporting an evidence-based classroom management program for preschoolers with disruptive behavior problems to a school: an analysis of implementation, outcomes, and contextual variables. *School Psychol. Quart.* 22 449–472. 10.1037/1045-3830.22.3.449

[B53] Singer J. D. Willett J. B. (2003). *Applied Longitudinal Data Analysis: Modeling Change And Event Occurrence.* London: Oxford University Press.

[B54] Statistics Norway. (2019). *Kindergartens.* Oslo: Statistics Norway.

[B55] Tveit H. H. Drugli M. B. Fossum S. B. Handegård H. Stenseng F. (2019). Does the incredible years teacher classroom management programme improve child–teacher relationships in childcare centres? A 1-year universal intervention in a Norwegian community sample. *Eur. Child Adoles. Psychiatry* 29 625–636. 10.1007/s00787-019-01387-5 31396707

[B56] Webster-Stratton C. Reid M. (2008). Adapting the incredible years child dinosaur social, emotional, and problem-solving intervention to address comorbid diagnoses. *J. Child. Serv.l* 3 17–30. 10.1108/17466660200800016

[B57] Webster-Stratton C. Reinke W. M. Herman K. C. Newcomer L. L. (2011). The incredible years teacher classroom management training: the methods and principles that support fidelity of training delivery. *School Psychol. Rev.* 40 509–529.

[B58] Werner C. D. Linting M. Vermeer H. J. Van IJzendoorn M. H. (2016). Do intervention programs in child care promote the quality of caregiver-child interactions? a meta-analysis of randomized controlled trials. *Prev. Sci.* 17 259–273. 10.1007/s11121-015-0602-7 26411312 PMC4718933

[B59] Zigler C. K. Ye F. (2019). A comparison of multilevel mediation modeling methods: recommendations for applied researchers. *Multi. Behav. Res.* 54 338–359. 10.1080/00273171.2018.1527676 30663388

